# Roles of TGF β and FGF Signals in the Lens: Tropomyosin Regulation for Posterior Capsule Opacity

**DOI:** 10.3390/ijms19103093

**Published:** 2018-10-09

**Authors:** Eri Kubo, Teppei Shibata, Dhirendra P. Singh, Hiroshi Sasaki

**Affiliations:** 1 Department of Ophthalmology, Kanazawa Medical University, Ishikawa 9200293, Japan; prefse74@yahoo.co.jp (T.S.); mogu@kanazawa-med.ac.jp (H.S.); 2 Department of Ophthalmology and Visual Science, University of Nebraska Medical Center, Omaha, NE 68198, USA; dpsingh@unmc.edu

**Keywords:** TGF β, tropomyosin, epithelial-mesenchymal transition, reactive oxygen species, FGF, peroxiredoxin 6, lens epithelium-cell derived growth factor

## Abstract

Transforming growth factor (TGF) β and fibroblast growth factor (FGF) 2 are related to the development of posterior capsule opacification (PCO) after lens extraction surgery and other processes of epithelial–mesenchymal transition (EMT). Oxidative stress seems to activate TGF β1 largely through reactive oxygen species (ROS) production, which in turn alters the transcription of several survival genes, including lens epithelium-cell derived growth factor (LEDGF). Higher ROS levels attenuate LEDGF function, leading to down-regulation of peroxiredoxin 6 (Prdx6). TGF β is regulated by ROS in Prdx6 knock-out lens epithelial cells (LECs) and induces the up-regulation of tropomyosins (Tpms) 1/2, and EMT of LECs. Mouse and rat PCO are accompanied by elevated expression of Tpm2. Further, the expression of Tpm1/2 is induced by TGF β2 in LECs. Importantly, we previously showed that TGF β2 and FGF2 play regulatory roles in LECs in a contrasting manner. An injury-induced EMT of a mouse lens as a PCO model was attenuated in the absence of Tpm2. In this review, we present findings regarding the roles of TGF β and FGF2 in the differential regulation of EMT in the lens. Tpms may be associated with TGF β2- and FGF2-related EMT and PCO development.

## 1. Introduction

The ocular lens is a transparent organ that changes its shape to adjust the focal distance of the eye in order to focus on objects. The crystalline lens consists of non-dividing central epithelial cells, dividing epithelial cells in the germinative zone, and terminally differentiated fiber cells in the bow region. Lens epithelial cells (LECs) maintain lens homeostasis and are not shed from the lens [1]. Damage to LECs is mediated by the abnormal growth of fiber cells, imbalances in the lens transport system, and the erroneous processing of biological molecules. There is compelling evidence that the progression of cataracts is associated with aging, and that oxidative stress is the major contributor to this process. Age-related cataracts are the primary cause of blindness in the world [2]. Age-related cataracts are a heterogeneous disorder that are related to multiple environmental and lifestyle risk factors that are modified by age, sex, exposure to solar ultraviolet (UV) radiation other types of radiation, oxidative stress, and cytokines [3,4,5]. Recently, growth factors have been analyzed to determine their roles in age-associated disorders including age-related cataracts. It has been reported that transforming growth factor (TGF) β is secreted in the aqueous and vitreous humors [6,7,8,9,10], and TGF β has been shown to induce cataractogenesis [11]. In addition, anterior subcapsular cataracts have been observed in lens-specific transgenic mice expressing TGF β1 [12]. These changes are also found in human cataracts. TGF β isoforms have been detected in mouse, rat, and human LECs (MLECs, RLECs, and HLECs, respectively) [13]. TGF β1 and TGF β2 mRNA are expressed primary in cultured HLECs [14]. Other cellular abnormalities, including the elevated expression of extracellular matrix proteins (ECM), have been attributed to reactive oxygen species (ROS)-related cell signaling [15]. Oxidative stress induces several functions, including cellular transformation and EMT. It is known that ROS mediates the expression and activation of TGF β. ROS down-regulates the survival pathway(s) in LECs, attenuates gene transcription, and overstimulates TGF β1-mediated signaling [16,17]. The TGF β1-mediated signaling pathway leads to the up-regulation of gene expression, including the genes for the *α smooth muscle actin* (*αSMA*) and *TGF-β-induced* (*βig-h3*) *protein*. Over-stimulation of these TGF-β-induced genes has been related to cataractogenesis, posterior capsular opacification (PCO), and myofibroblast-related disorders [5,9,17].

**Figure 1:**
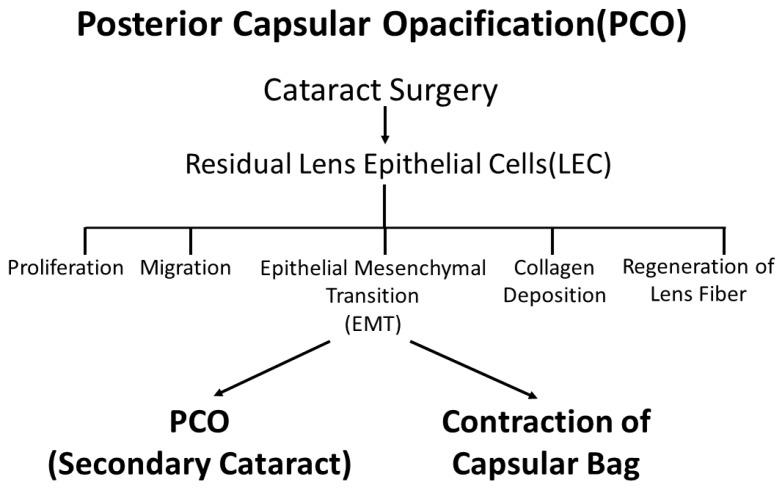
Changes in LECs observed in PCO. After cataract surgery, residual LECs induce cell proliferation, migration, EMT, collagen deposition and regeneration of lens fibers causing PCO, or so-called secondary cataracts.

**Figure 2:**
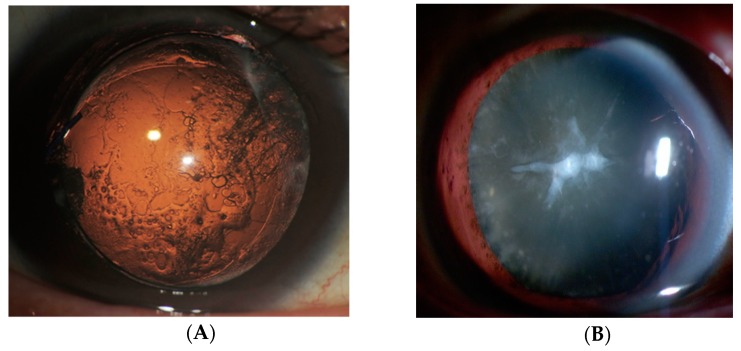
PCO and cataracts with Anterior subcapsular fibrosis (ASF). (**A**) Slit-lamp retroillumination photos of PCO development. PCO is the most common complication of cataract surgery. It is caused by the migration of residual LECs from the equatorial region of the lens capsule. (**B**) A slit-lamp image of a cataract with ASF. Cataracts with ASF are usually seen at the anterior pole of the lens. Fibroblastic LECs are observed in this portion.

PCO is a common, significant complication following cataract surgery. At present, surgical intervention is the only cure [18]; however, after cataract surgery, the growth of aberrant LECs across the lens capsule often leads to migration, fibrosis and collagen deposition. This results in secondary visual loss known as PCO, which is sometimes referred to as a “secondary cataract” or “after cataract” [19] (Figure 1 and Figure 2A). Capsular phimosis is also caused by excessive anterior capsular fibrosis. Epithelial-mesenchymal transition (EMT) is a major cause of the progression of several eye diseases, including PCO, cataracts with anterior subcapsular fibrosis (ASF), also known as anterior subcapsular cataracts (Figure 2B), pterygium, and glaucoma, and is part of the wound-healing process post-ocular surgery [18,20,21,22,23,24,25,26,27]. Aberrant TGF β signaling plays a major role in the EMT of cells and tissues akin to its role in the development of human ASF [4,28,29,30] and PCO [12,18,30,31,32]. TGF β promotes tissue fibrosis, transdifferentiation, myofibroblast formation, EMT, fibrosis, cell proliferation, and apoptosis [33,34], by up-regulating genes such as αSMA and tropomyosins (Tpms), which are implicated in a variety of pathogenic processes, including cataractogenesis [35,36]. This molecular event may be a prime cause of ASF [4,28,30] and PCO [11,12,29,30,31]. ASF results from abnormal proliferation and myofibroblastic changes in LECs to form white plaques that obscure vision [37]. ASF can be caused by ocular trauma and inflammatory diseases including post-intraocular surgery, atopic dermatitis and uveitis [38].

Previous studies have suggested that fibroblast growth factor (FGF) also contributes to PCO development [39,40]. The concentration of FGF is higher in the vitreous chamber than the anterior chamber [41]. FGF also initiates the differentiation of LECs into fiber cells in vivo [41]. In in vitro experiments using lens epithelial explants, FGF has been shown to induce lens fiber cell elongation and differentiation [42]. FGF also induces many other cellular changes, such as cell elongation, the loss of organelles, and denucleation in the bow region of lens fibers [43,44,45,46]. In transgenic mice that over-express FGFs and FGF receptors, the normal development and growth of the lens are also impaired [47,48,49], indicating that FGF plays an important role in lens development and differentiation [50]. FGF2 is expressed in HLECs [51] and is involved in regulating cell proliferation and migration during lens development [52]. This molecule induces dose-dependent cell proliferation, lens fiber differentiation, and formation of collagen in LECs [44,45,50,53]. FGF2 also reduces the contraction of LEC-containing collagen gel in vitro and decreases the expression of αSMA in LECs [36], indicating that FGF2 reduces fibroblast function and has an effect opposite to that of TGF β in the wound healing process. Despite the many studies on the roles of TGF β in the lens, the mechanism underlying the synergistic action of TGF β and FGF2 in EMT remains unclear. Moreover, the role of these molecules in gene expression, including the expression of cytoskeleton proteins such as αSMA and Tpms during PCO development, has not been elucidated.

## 2. Activation of TGF β Is Associated with ROS, and TGF β Generates ROS via the Down-Regulation of Peroxiredoxin 6 (Prdx6)

Several mechanisms for TGF β activation have been reported [54,55], including proteases, trauma, integrin, and ROS [56]. In addition, TGF β1 has been shown to increase ROS generation through the down-regulation of antioxidant-related genes such as *catalase*, *Mn-superoxide dismutase* and *lens epithelium-derived growth factor* (*LEDGF*) [17,57]. Cloned *LEDGF* is a survival factor and transcriptional activator [58]. *LEDGF* has been shown to activate cell proliferation at low concentrations. Cells expressing higher concentrations of LEDGF exhibit enhanced survival against many types of stresses [59,60,61,62]. In particular, stresses such as thermal and oxidative stress increase the expression of *LEDGF* mRNA and proteins [63]. Our previous study revealed that TGF β1 treatment markedly decreased *LEDGF*, *heat shock protein* (*Hsp*) *27*, and *αB-crystallin* promoter activities [58]. TGF β1 induces the down-regulation of *LEDGF* expression and reduces the DNA binding affinity in HLECs that exhibit fibroblastic changes and apoptosis [60]. TGF β1 suppressed the function of *LEDGF* by reducing its transcription and inhibiting its DNA binding [58]. Previous studies have reported that TGF β plays a regulatory role in the transcription of genes containing TGF β inhibitory elements (TIE) [64,65]. Our experiments established that TIE is present in the *LEDGF* promoter [58]. The interruption of a TIE element, nnnnTTGGnnn, found at positions –444 to –433 in the *LEDGF* promoter, suppressed the TGF-β1-induced inhibition of *LEDGF* expression [58]. Thus, the repression of *LEDGF* may be a critical event in TGF-β1-induced changes [58].

LEDGF activates a heat shock element (HSE; nGAAn) and a stress-related element (STRE; T/AGGGGA/T) in stress-related genes such as *Hsp25*, *Hsp 27*, *Hsp90*, *αB-crystallin*, and *Prdx6* [63,66,67]. The over-expression of *LEDGF* in cells induces the up-regulation of the Prdx6 protein and mRNA, and increases cell survival under oxidative and heat stress conditions [63]. Higher ROS insults attenuate the function of LEDGF in cells and subsequently lead to the decreased production of Prdx6 [63]. Six members of the Prdx family have been identified to date. Prdxs use redox-active cysteine (Cys or C) to reduce peroxides [68]. Prdx6 protects cells from environmental stresses. Notably, our report revealed the presence of all six known Prdxs in the lens; however, Prdx6 was expressed at a higher level than the others [69]. This finding indicates that the expression levels and functions of Prdxs may be tissue-specific. Moreover, several lines of evidence now demonstrate that Prdx6 is important in maintaining cellular homeostasis by controlling ROS and modulating gene regulation. In addition, Prdx6 plays an important role in cellular signaling by reducing ROS and thereby regulating gene expression [17,63,69,70,71]. 

We have shown that Prdx6-deficient (*Prdx6^−/−^*) MLECs are more vulnerable to UV-triggered cell death, and these cells display abnormal protein profiles [72]. We found that ROS levels are elevated in *Prdx6^−/−^* cells as well as in aged LECs [17]. Furthermore, levels of activated TGF β1 are increased in *Prdx6^−/−^* LECs. The addition of Prdx6 abolished the increase of activated TGF β1, indicating that the ROS-induced activation of TGF β1 is the major cause of EMT-like morphological changes in *Prdx6^−/−^* LECs [17]. In our previous studies, the expression of Prdx6 was reduced and the expression of *TGF β1* and *aSMA* was markedly up-regulated in cataractous lenses from Shumiya cataract rats [73]. In addition, intraconjunctival injection of Prdx6 recombinant protein in the eyes of Shumiya cataract rats delayed the progression of lens opacity [73]. Bioactive TGF β1 is also known to be present in the aqueous humor and vitreous body in humans and other species [74,75]. ROS promote the expression and activation of TGF β, which suppresses the survival pathway(s) in LECs by negatively regulating the transcription of genes such as *LEDGF* [17]. ROS-TGF β signaling induces phenotypic changes in LECs and up-regulation of *aSMA*, which are similar to the effects observed in human ASF and PCO. Several previous reports also demonstrated that the activation of TGF β1 is enhanced by ROS elevation [17,56,76,77,78]. ROS-driven abnormal signaling overstimulates TGF β1-mediated signaling [16,17,79]. This leads to over-modulation of the expression of certain genes, such as *LEDGF*, *Prdx6*, *αSMA*, *Tpm*, and *βig-h3*, changes in which have been implicated in the induction of cataracts, ASF and PCO as well as other EMT-related disorders [5,9,17,80].

## 3. *Tpm* and Various ECM-Related Genes are Highly Up-Regulated in *Prdx6* Depletion in LECs

In *Prdx6^−/−^* LECs, phenotypic changes such as the development of EMT-like morphology have been associated with the formation of lentoid bodies, a characteristic of the terminal cell differentiation of LECs [72]. To identify the proteins involved in the responses to *Prdx6* depletion in MLECs, we used a proteomic differential display method, fluorescence-based difference gel electrophoresis (DIGE), coupled with matrix-assisted laser desorption/ionization time-of-flight (MALDI-TOF) mass spectrometry (MS) [72]. Comparative proteomic analysis revealed the elevated expression of cytoskeleton proteins such as Tpm1, Tpm2, Vimentin, and TGF β1 [72]. Protein blot and real-time PCR validated marked increases in Tpm1 and Tpm2 expression in these cells. The expression of *Tpm2* mRNA was extremely low in the LECs and lenses; however, the *Tpm*2 mRNA and proteins in *Prdx6^−/−^* MLECs were highly up-regulated compared to those in wild-type MLECs [80]. We therefore hypothesized that, because Tpms have been shown to play a role in the assembly of actin filaments and the control of cell motility via the stabilization of actin filaments, aberrant expression of Tpm2 may be related to phenotypic changes in *Prdx6^−/−^* MLECs [80]. The actin cytoskeleton plays a significant role in the regulation of cells linked to PCO. Dysregulation of Tpm isoforms is expected to be a major indicator of EMT-like cellular changes and PCO [81,82]. The human *Tpm* genes were designated as *TPM1* through *TPM4* (*Tpm1* through *Tpm4* for mouse and rat *Tpm*) [83]. The various Tpm isoforms under each gene were generated via alternative exon splicing [83,84]. The levels of Tpm isoforms in the cells altered each of the Tpm functions [82,85,86,87]. Increased expression of Tpm isoforms, such as the *Tpm1* and *Tpm2* genes in MLECs, indicated that these isoforms play a role in the EMT-like morphological alteration of *Prdx6^−/−^* MLECs. Importantly, a similar pattern of Tpm2 protein expression after TGF β1 or H_2_O_2_ treatment was found in wild-type MLECs [35,72]. Further, the addition of exogenous Prdx6 into *Prdx6^−/−^* LECs attenuated the up-regulation of Tpm2 expression. Thus, the phenotypic alterations displayed in *Prdx6^−/−^* MLECs may be due to the aberrant expression of the *Tpm1* and *Tpm2* genes derived from ROS-induced activation of TGF β. It has been reported that TGF β specifically up-regulates the expression of the *Tpm1* and *Tpm2* genes, which encode low-molecular-mass *Tpms* [86,88]. Thus, Prdx6 may attenuate adverse ROS -TGF β signaling, thereby reducing oxidative stress in LECs, and ultimately resulting in the maintenance of cellular homeostasis.

## 4. Elevated Tpm Expression Is Associated with EMT of LECs in PCO and Human Cataracts with ASF

We assessed the expression of isoforms from the *Tpm1* and *Tpm2* genes in a mouse and a rat model of PCO in vivo [80,89], and in LECs obtained from human subjects of various ages with cataracts [80]. Extracapsular lens extraction (ECLE) surgery was performed in the eyes of mice and rats to induce PCO. Under normal physiological conditions, the expression of Tpm1 and Tpm2 in LECs was minimal in mice and rats in vivo [80]. However, we found that the expression of Tpm1 and Tpm2 markedly increased during the development of EMT-like fibroblastic changes in LECs obtained from mice and rats with PCO, and demonstrated that the selective elevation of Tpm1 and Tpm2 in mouse and rat LECs compared to controls was correlated with EMT in the remaining LECs after ECLE [80,89]. The expression of αSMA, a marker of the differentiation and EMT of LECs, was elevated and co-localized with Tpm1 and Tpm2 in the rat with PCO [80], suggesting that the expression of Tpm1 and Tpm2 may be associated with the progression of EMT-like changes observed in PCO. Activation of TGF β may be induced by inflammation, mechanical stress and ROS during intraocular surgery, and would in turn induce the wound healing process and EMT by up-regulating the *Tpm1* and *Tpm2* genes, finally leading to PCO [80]. Moreover, we have reported that the expression of Tpm1 and Tpm2 was induced/elevated in the HLECs of human cataracts with ASF, or so-called anterior subcapsular cataracts, and in human PCO tissues surgically removed from patients affected by non-traumatic dislocated intraocular lens (IOL) [80]. Previous analysis of capsular bags from human donor eyes that received cataract surgery with IOL revealed a high level of endogenous active TGF β2, matrix wrinkling of the lens capsule, and increased expression of αSMA and fibronectin [13]. The induction of Tpm isoforms and stress fibers in mouse and rat PCO and LECs has been suggested to play a major role in the TGF β regulation of myofibroblast formation and wrinkling of the lens capsule, and may be necessary for the TGF β-mediated formation of stress fibers [80,86]. It has been reported that *Tpm1* and *Tpm2* are responsible for the assembly of stress fibers and are targeted by TGFβ [86,88]. Tpms have been shown to play a significant role in stress fiber formation by stabilizing actin filaments [35,90]. TGF β specifically induces the expression of *Tpm1* and *Tpm2*, but is not involved in the regulation of either *Tpm3* or *Tpm4* [86,88]. Tpm knock-down by the transfection of siRNA against Tpm1 and Tpm2 completely blocked TGF β-induced stress fiber formation in cultured mouse epithelial NMuMG and human cervical carcinoma SiHa cells, whereas the over-expression of Tm1.6 and Tm1.7 isoforms, derived from the *Tpm1* gene, induced stress fibers even without addition of TGF β or other cytokines in human breast cancer MDA-MB-231 cells [86]. EMTs of LECs have been observed in ASF and PCO [19,91,92]; these changes may be affected by the TGF β-induced induction of *Tpm1* and *Tpm2*. Regardless of the pathophysiological importance of Tpm isoforms, we suggest that the distinct pattern of *Tpm* expression may function as a clinical marker of LEC differentiation, PCO or ASF.

## 5. TGF β and FGF2 Regulate the EMT of LECs and the Expression of Tpms

**Figure 3:**
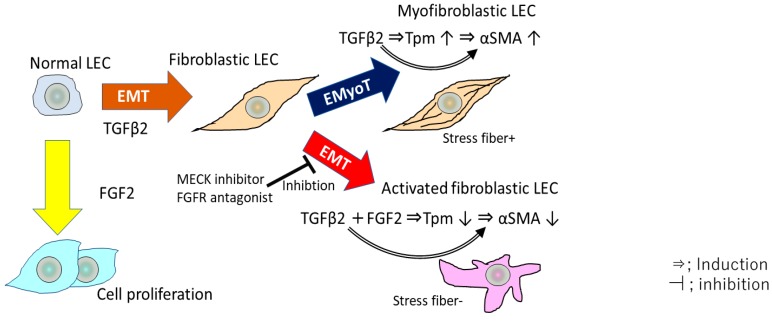
Overview of EMT and EMyoT induction in LECs by TGF β2 and FGF2. TGFβ induces EMyoT and Tpm expression. Tpms promote the formation of stress fibers undergoing EMyoT. FGF2 suppresses the TGFβ2-induced over-expression of Tpms. Depletion of Tpms by FGF suppresses the formation of stress fibers and activates fibroblastic LECs, which lose cell polarity, inducing cell migration.

In an in vitro study using cultured MLECs and HLECs, we showed that Tpm1 and Tpm2 are involved in regulating and stabilizing actin microfilaments (F-actin) in MLECs [35]. TGFβ induces the epithelial-to-myofibroblastic transition (EMyoT) in the LECs, which is observed in the up-regulation of Tpm and αSMA, and in the formation of F-actin. Importantly, Tpms may induce the formation of stress fibers undergoing EMyoT. However, we have shown that FGF2 suppresses the TGFβ2-induced expression of Tpm1, Tpm2 and αSMA, resulting in the recession of stress fiber formation and thereby the activation of myofibroblastic LECs, which promote cell migration [35] (Figure 3). Stress fiber formation and up-regulation of αSMA induced by TGF β2 could be reversed by transfection of siRNA against the *Tpm1* and *Tpm2* genes [35], suggesting that *Tpms* may be necessary to organize stress fibers in response to TGFβ2 during the EMT process. Furthermore, the expression of Tpm1 and Tpm2 and the formation of stress fibers induced by TGF β2 could be inverted by FGF2 addition in MLECs and HLECs [35]. We found that normal MLECs exhibited enhanced migration in response to combined TGF β and FGF2 stimulation [35]. Previous studies have examined in detail the major role played by FGF2 in the PCO process [1,27,72]. FGF2 and its family members are known to play an important role in the development and maintenance of normal lens differentiation, growth and function [27]. The FGF2 concentration in the eyes may increase after cataract surgery, stimulating cell proliferation and differentiation [72,73]. In a previous study using a rabbit PCO model, the level of active TGFβ decreased immediately after cataract surgery, then returned to a normal level sufficient to stimulate EMT at about two weeks post-surgery [35]. Similarly, a previous study has shown the dimension of EMT induced by TGF β in cooperation with FGFs in cancer cell lines [93]. FGF2 repressed TGF β-induced EMyoT and enhanced the EMT, conferring greater invasiveness similar to that of activated fibroblasts in cancer cell lines [93]. Moreover, the EMT-induced cells treated with FGF2 and TGF β promoted cancer cell invasion [93]. During the EMT process, isoforms of FGF receptors were switched by TGF β treatment, causing the cells to be more reactive to FGF2 [93]. Therefore, TGF β and FGF2 reciprocally collaborate and may regulate the EMT in the cancer microenvironment [93] and in LECs during PCO development. 

Many studies have reported that FGF is a major activator of the mitogen-activated protein kinase/extracellular signal–regulated kinase (MAPK/ERK) 1/2 pathway in the eye lens in vivo [94,95]. In our study, FGF2 delivery to TGF β-treated LECs agitated the EMyoT by reactivating the MAPK/ERK pathway and subsequently enhancing the EMT and cell migration and repressing stress fiber formation [35]. Loss of Tpms induced by FGF2 and TGF-β co-treatment was significantly linked to the MAPK/ERK1/2 pathway in MLECs. Conversely, a mitogen-activated protein kinase kinase (MEK) inhibitor (PD98059) and FGFR antagonist (SU5402) abated FGF2-mediated Tpm1/2 and αSMA suppression [35]. These results support our understanding of the mechanism and signaling for the reprogramming of the actin cytoskeleton during EMT, cell proliferation, and invasion of LECs in PCO development and progression. Therefore, we propose that clarifying the physiological link between the levels of FGF2, Tpm1 and Tpm2 expression, and TGF β-orchestrated EMyoT may help to develop therapeutic targets based on Tpm1 and Tpm2 to prevent PCO (Figure 3).

## 6. *Tpm2* Knock-Down Reduced Wound Healing of the Mouse Lens In Vivo

Upon injury to the anterior capsule, LECs transdifferentiate into fibroblastic cells during the healing process, or so-called EMyoT [96,97], and transform into myofibroblasts positive for αSMA, an established marker of this process. Thus, we used this injury-induced EMT in the lens capsule as a mouse PCO model. To evaluate the physiological role of Tpm2 in PCO, we generated *Tpm2* heterozygous knockout mice using clustered regularly interspaced short palindromic repeats/Cas9 (CRISPR/Cas9). Mice with homozygous loss of *Tpm2* were embryonically lethal, suggesting that *Tpm2* is necessary for embryonic development and life [89]. Previous studies have also reported that the *Tpm1-* and *3*-knockout mouse models were embryonically lethal [98,99,100]. Therefore, *Tpm2* heterozygous knockout (*Tpm2^+/−^*) mice were analyzed as an animal model in which the expression of *Tpm2* was reduced. *Tpm2^+/^^−^* did not change the expression of *Tpm1* or *αSMA* in the lens. Therefore, we suggest that the abnormal phenotype observed in *Tpm2^+/−^* mice is mostly due to *Tpm2* gene heterozygous deletion [89]. To induce EMT in the lens as a kind of PCO model, a wound healing model was prepared in seven-week-old mice following a previously reported technique with modifications [97]. In brief, a wound was generated by puncturing the central anterior lens capsule with a 31-gauge needle [89]. Wounded areas at the lens surface showed fibroblast-like tissue changes, indicating EMT [89]. The fibroblastic, EMT/EMyoT-like changes observed in the wounded area were less severe in the *Tpm2^+/^^−^* mice than in the wild-type mice, suggesting that the depletion of *Tpm2* may suppress wound healing in the mouse lens. These results suggest that Tpm2 may play a significant role in the initiation and progression of EMT/EMyoT in the lens [89]. Moreover, immunolocalization of αSMA and Tpm2 is reduced in the wounded area of the lens in *Tpm2^+/^^−^* mice. Thus, these results revealed that knock-down of *Tpms* may suppress the induction of EMT/EMyoT in wound healing and PCO.

## 7. Conclusions

ROS up-regulate TGF β-mediated signaling, inducing the aberrant expression of certain genes, including those encoding *Tpm1*, *Tpm2*, *LEDGF*, and *αSMA*, which are involved in the induction of EMT in LECs [17,35,58]. We identified two types of TGFβ2-induced transdifferentiation of LECs with or without FGF2. One type was transdifferentiated to TGFβ-induced Tpm- and αSMA-positive myofibroblastic cells exhibiting EmyoT-like morphology unaccompanied with FGF2, and the other was transdifferentiated to FGF2-induced Tpm- and αSMA-negative fibroblastic cells observed in EMT accompanied with TGF β2 treatment [35]. Elevation in Tpm expression may be related to the progression of EMyoT in mouse and rat PCO and wound healing in MLECs [35,89]. Tpms may act as important biomarkers and therapeutic targets in the treatment of PCO, in the wound healing process and in cancer invasion. Further, we expect that elucidation of the mechanism of Tpm regulation will provide helpful information for the generation of inhibitors of Tpm1 and Tpm2, and ultimately for the treatment and prophylaxis of EMT-related diseases.

## Abbreviations

LECs	Lens epithelial cells
UV	Ultraviolet
TGF	Transforming growth factor
MLECs	Mouse LECs
RLECs	Rat LECs
HLECs	Human LECs
ECM	Extra cellular matrix protein
ROS	Reactive oxygen species
αSMA	α-Smooth muscle actin
βig-h3	TGF-β-induced protein
PCO	Posterior capsule opacification
EMT	Epithelial-mesenchymal transition
ASF	Anterior subcapsular fibrosis
Tpms	Tropomyosins
FGF	Fibroblast growth factor
Prdx	Peroxiredoxin
LEDGF	Lens epithelium-cell derived growth factor
Hsp	Heat shock protein
TIE	TGF β inhibitory elements
*Prdx6^-/-^*	Prdx6-deficient
DIGE	Difference gel electrophoresis
MALDI-TOF	Matrix-assisted laser desorption/ionization time-of-flight
MS	Mass spectrometry
ECLE	Extracapsular lens extraction
IOL	Intra ocular lens
EMyoT	Epithelial-to-myofibroblastic transition
MAPK/ERK	Mitogen-activated protein kinase/extracellular signal–regulated kinase
MEK	Mitogen-activated protein kinase kinase
CRISPR/Cas9	Clustered regularly interspaced short palindromic repeat/Cas9
*Tpm2^+/-^*	Tpm2 heterozygous knockout
